# Simple paired heavy- and light-chain antibody repertoire sequencing using endoplasmic reticulum microsomes

**DOI:** 10.1186/s13073-018-0542-5

**Published:** 2018-04-27

**Authors:** Praneeth Reddy Devulapally, Jörg Bürger, Thorsten Mielke, Zoltán Konthur, Hans Lehrach, Marie-Laure Yaspo, Jörn Glökler, Hans-Jörg Warnatz

**Affiliations:** 1 Otto Warburg Laboratory Gene Regulation and Systems Biology of Cancer, Max Planck Institute for Molecular Genetics, Berlin, Germany; 2 Microscopy and Cryo-Electron Microscopy Service Group, Max Planck Institute for Molecular Genetics, Berlin, Germany; 3 Institut für Medizinische Physik und Biophysik, Charité-Universitätsmedizin, Berlin, Germany; 4 Department of Biomolecular Systems, Max Planck Institute of Colloids and Interfaces, Potsdam, Germany; 5 Alacris Theranostics GmbH, Berlin, Germany; 6 Dahlem Centre for Genome Research and Medical Systems Biology, Berlin, Germany; 7 Department of Molecular Biotechnology and Functional Genomics, Institute of Applied Biosciences, Technical University of Applied Sciences Wildau, Wildau, Brandenburg Germany

## Abstract

Existing methods for paired antibody heavy- and light-chain repertoire sequencing rely on specialized equipment and are limited by their commercial availability and high costs. Here, we report a novel simple and cost-effective emulsion-based single-cell paired antibody repertoire sequencing method that employs only basic laboratory equipment. We performed a proof-of-concept using mixed mouse hybridoma cells and we also showed that our method can be used for discovery of novel antigen-specific monoclonal antibodies by sequencing human CD19^+^ B cell IgM and IgG repertoires isolated from peripheral whole blood before and seven days after Td (Tetanus toxoid/Diphtheria toxoid) booster immunization. We anticipate broad applicability of our method for providing insights into adaptive immune responses associated with various diseases, vaccinations, and cancer immunotherapies.

**Electronic supplementary material:**

The online version of this article (10.1186/s13073-018-0542-5) contains supplementary material, which is available to authorized users.

## Background

High-throughput sequencing of immunoglobulin repertoires from B cells has emerged as a powerful tool to investigate repertoire changes for antibody discovery, vaccine efficacy studies, and in other healthcare applications [1–3]. Initially, antibody repertoire analysis focused on obtaining information from antibody heavy chains (HC) only [4–7], missing the native light-chain (LC) pairing information that is necessary for antibody cloning and expression. Retaining paired HC-LC data from bulk B cell populations at single-cell level remained a major obstacle for a long time. To this end, several single-cell paired sequencing technologies were reported more recently, which were initially limited by low cell numbers (< 400–10^5^ cells) and sometimes required the use of complex microfluidic systems [8–12]; however, cellular throughput is improving through newer developments, such as droplet-based systems and the 10× Genomics platform [13, 14]. More recently, two emulsion-based methods reported paired HC-LC repertoire sequencing from 2–3 × 10^6^ B cells at single-cell level [15, 16]. Although substantial, the existing methods are limited by their commercial availability, high costs, and require an elaborate construction of flow-focusing or microfluidic devices and dedicated personnel for operation [17]. Here, we describe a high-throughput method which enables sequencing of paired HC-LC immunoglobulin (Ig) repertoires from millions of B cells simply by using a cooled table-top centrifuge, a magnetic stirrer, and a thermal cycler. This method makes paired Ig sequencing widely applicable even for laboratories without specialized equipment and personnel.

## Methods

### Cell lines

The HEK 293T cell line was obtained from the American Type Culture Collection (ATCC CRL-3216). Mouse hybridoma cell lines KT13 and KT22 were obtained from the Developmental Studies Hybridoma Bank (DSHB). Both cell lines were deposited to the DSHB by Kazumasa Takeda and Asako Sugimoto (DSHB hybridoma products KT13 and KT22). Mouse hybridoma cell line 5E4/1F1 was kindly provided by Miha Kosmač and Vladka Čurin Šerbec (University of Ljubljana). HEK 293T and hybridoma cells were grown in DMEM (Gibco) supplemented with 13% FBS (Gibco), 1× Penicillin/Streptomycin (Thermo Fisher), and 1× GlutaMAX (Gibco). Antibody HC and LC sequences from individual hybridomas were determined by reverse transcription polymerase chain reaction (RT-PCR) and capillary sequencing (Eurofins Genomics).

### Cycloheximide treatment and microsome preparation

All pipetting steps were performed on ice and centrifugations were carried out at 4 °C using an Eppendorf 5810R centrifuge with fixed angle rotor F-45-30-11. Protein LoBind 1.5 mL centrifuge tubes (Eppendorf) were used to minimize cell adhesion to the tube walls. HEK 293T cells (1 million), mouse hybridoma cells (1 million of 5E4, KT13, and KT22 cells mixed in ratio 1:1:1), ARH-77 leukemia cells (ATCC CRL-1621, 1 million), or freshly isolated human CD19^+^ B cells from pre- and post Td-booster immunization samples (1.5 million each) were resuspended in 1 mL PBS with 50 μg/mL cycloheximide and incubated for 10 min to stall ribosomes with associated messenger RNAs (mRNA) at the rough endoplasmic reticulum. The cells were pelleted with 300 g for 10 min at 4 °C and resuspended by pipetting 15× up and down in 120 μL high-density lysis buffer (25 mM HEPES-KOH pH 7.2, 110 mM potassium acetate, 5 mM magnesium acetate, 1 mM EGTA, 25% [w/w] sucrose [0.81 M], 5% [v/v] glycerol, 1 mM 1,4-dithiothreitol, 1× cOmplete EDTA-free protease inhibitor cocktail [Roche], 0.1 mg/mL cycloheximide, 0.015% digitonin, and 400 U/mL RiboLock RNase inhibitor [Thermo Fisher Scientific]). Cell and organelle lysis was completed by incubation for 10 min on ice. Each homogenate was split into two 55 μL aliquots and transferred into two fresh Protein LoBind tubes. The tubes were centrifuged at 600 g for 3 min at 4 °C to pellet nuclei and cell debris. A total of 40 μL supernatant from each tube, containing membrane fractions and cytosol, were transferred into fresh Protein LoBind tubes and the sucrose concentration was diluted to 0.37–0.40 M (12–13% w/w) by the addition of 40 μL nuclease-free water. Microsomes were then sedimented by centrifugation with 20,800 g for 120 min at 4 °C. The cytosol-containing supernatants were discarded and membrane pellets were resuspended by pipetting 10× up and down in 85 μL wash buffer (25 mM HEPES-KOH pH 7.2, 110 mM potassium acetate, 2.5 mM magnesium acetate, 1 mM EGTA, 1 mM 1,4-dithiothreitol, 1× cOmplete EDTA-free protease inhibitor cocktail, 0.1 mg/mL cycloheximide, 0.004% digitonin, and 400 U/ml RiboLock RNase inhibitor). The microsomes were sedimented again by centrifugation with 20,800 g for 60 min at 4 °C. Supernatants were discarded and the microsome pellets were resuspended in 20 μL of wash buffer and kept on ice until further use.

### Transmission electron microscopy

Sample aliquots of 3.5 μL of resuspended HEK 293T microsomes were applied to freshly glow-discharged Quantifoil grids (Quantifoil, Germany) covered with an additional 2 nm carbon support film and flash-frozen in liquid ethane using a Vitrobot plunger (FEI). Samples were imaged on a Tecnai Spirit transmission electron microscope (FEI) operated at 120 kV equipped with a 2 × 2 k Eagle CCD camera (FEI). Micrographs were recorded under cryo low-dose conditions at 42,000× nominal magnification (pixel size at object scale: 5.2 Å/px) applying a defocus of − 2 to − 4 μm. Data collection was performed either manually or fully automatically using Leginon [18].

### Emulsion RT-PCR assembly using mouse hybridoma microsomes

We diluted 16 μL of resuspended microsomes from mixed hybridomas 5E4, KT13, and KT22 in 184 μL RT-PCR master mix containing 1× Verso 1-Step RT-PCR master mix (Thermo Scientific), 1× Verso enzyme mix (Thermo Scientific), 0.5 μg/μL BSA, 100 μg/mL cycloheximide, and primers for reverse transcription and HC and LC assembly (0.8 μM each of primers TitA_MID1_IgM_rev and TitB_MID12_IgK_rev; 0.16 μM each of primers OE_MHV_fwd and OE_MKV_fwd). Primer sequences are shown in Additional file 1: Figure S1a. The resulting 200 μL of aqueous solution were used to form a water-in-oil emulsion by dropwise addition (13 aliquots of 15 μL in 30-s intervals) to 800 μL oil phase according to Ge et al. [19] (Mineral oil, Sigma M5904, with 4.5% [v/v] Span 80, Sigma S6760, 0.4% [v/v] Tween 80, Sigma P8074, and 0.05% [v/v] Triton X-100, Sigma T8787) during continuous stirring on a magnetic stirrer. Six aliquots of 100 μL each of the resulting emulsion were transferred into PCR tubes and subjected to thermocycling with the following conditions: reverse transcription at 50 °C for 15 min, RTase inactivation at 95 °C for 2 min, then four cycles of denaturation at 95 °C for 20 s, annealing rampdown from 60 °C to 50 °C for 50 s and extension at 72 °C for 1 min, then 16 cycles of denaturation at 95 °C for 20 s, annealing at 60 °C for 30 s and extension at 72 °C for 1 min, followed by a final extension step at 72 °C for 5 min. In parallel, an open RT-PCR control was performed by diluting 4 μL of resuspended microsomes in 46 μL RT-PCR master mix and thermocycling the reaction in parallel with the emulsion RT-PCR. PCR assembly products were extracted from the emulsion using isobutanol (2-Methyl-1-propanol, Sigma) and the Zymo DNA Clean & Concentrator-5 kit (Zymo Research) as previously published [20]. The resulting DNA and the PCR product from the open PCR control were loaded on a 1.2% TBE-agarose gel and separated with 90 V for 60 min. Assembly products of 800–950 bp size were size-selected from the agarose gel and the products were recovered using a Zymoclean Gel DNA Recovery kit. Assembly products were eluted in 6 mM Tris-Cl pH 8 and stored at − 20 °C until further analysis.

### Nested PCR amplification of mouse hybridoma assembly products

After the emulsion assembly reaction, the assembly products were further amplified with adapter primers TitA_fwd, 5’ CGT ATC GCC TCC CTC GCG CCA TCA G 3′, and TitB_rev, 5’ CTA TGC GCC TTG CCA GCC CGC TCA G 3′, using the Phusion high-fidelity DNA polymerase kit (Finnzymes) with the following thermocycling conditions: Initial denaturation at 98 °C for 30 s, then 15 cycles of denaturation at 98 °C for 7 s and annealing/extension at 72 °C for 30 s, followed by a final extension step at 72 °C for 5 min. PCR products were purified with the Zymo DNA Clean & Concentrator-5 kit. The pairing of HC and LC in the assembly products were then analyzed by PCR using nested primers specific for the three different HC and three different LC (Additional file 1: Figure S1e) using the Phusion high-fidelity DNA polymerase kit with the following thermocycling conditions: initial denaturation at 98 °C for 30 s, then 24 cycles of denaturation at 98 °C for 7 s and annealing/extension at 72 °C for 30 s, followed by a final extension step at 72 °C for 5 min. Nested PCR products were loaded on a 1.2% TBE-agarose gel and separated with 90 V for 40 min. Real-time nested PCR for quantification of cross-contamination was carried out in triplicates with the same nested primers using SYBRGreen master mix (Applied Biosystems) on a StepOne qPCR cycler (Applied Biosystems) with the following thermocycling conditions: initial denaturation at 95 °C for 10 min, followed by 40 cycles of denaturation at 95 °C for 15 s, annealing at 56 °C for 30 s and extension at 72 °C for 45 s. The initial abundances of the amplified assembly products were calculated using the 2^(-deltaCt) method and plotted as bar charts with error bars showing standard deviation from the mean.

### Immunization and CD19^+^ B cell isolation from peripheral whole blood samples

The human peripheral whole blood samples used in this study were obtained from in.vent Diagnostica GmbH as by-products from routine diagnostic procedures. in.vent Diagnostica GmbH has written informed consent from the donor to use the by-products for research and has an ethical approval from the Freiburg Ethics Commission International (FEKI code 011/1763) for the distribution of samples. A healthy proband underwent booster immunization with Tetanus Toxoid (TT)/Diptheria Toxoid (DT) (Td-pur®; 20 International units [IU] TT and 2 IU DT; Novartis, Basel, Switzerland). K2-EDTA peripheral whole blood derived from pre-immunization (day 0) and seven days post-Td booster immunization were used to isolate CD19^+^ B cells using the CD19 pluriBead Cell Separation Kit (pluriSelect GmbH, Leipzig, Germany) following the manufacturer’s protocol. Isolated CD19^+^ B cell pellets were washed in 1 mL cold PBS and centrifuged at 300 g for 10 min at 4 °C. Cell pellets corresponding to 1.5 million B cells from both pre- and post-immunization samples were kept on ice until cycloheximide treatment and microsome preparation.

### Emulsion RT-PCR assembly using human B cell microsomes

We added 2 μL of diluted microsomes prepared from frozen ARH-77 cells (as internal pairing control) to 26 μL of resuspended microsomes from B cells both pre- and post-Td immunization, so that the final fraction of ARH-77 microsomes is 0.5% (v/v). We diluted 16 μL of this microsomes suspension in 184 μL RT-PCR master mix containing 1× dART 1-step RT-PCR master buffer mix (Roboklon), 2× dART master enzyme mix (Roboklon), 0.5 μg/μL BSA, 100 μg/mL cycloheximide and primers for reverse transcription (IgM, IgG, and IgK) and heavy (VH) and light chain (VK) assembly. Primer sequences and concentrations in the RT-PCR master mix are listed in Additional file 1: Table S2. The resulting 200 μL of aqueous solution were used to form a water-in-oil emulsion by dropwise addition (13 aliquots of 15 μL in 30 s intervals) to 800 μL oil phase composed of 73% emulsion component 1, 7% emulsion component 2, and 20% emulsion component 3 of the Micellula DNA emulsion and purification kit (Roboklon) during continuous stirring on a magnetic stirrer. Six aliquots of 100 μL each of the resulting emulsion were transferred into PCR tubes and subjected to thermocycling with the following conditions: Reverse transcription at 55 °C for 30 min, initial denaturation at 95 °C for 3 min, then three cycles of denaturation at 95 °C for 20 s, annealing at 56 °C for 30 s and extension at 72 °C for 2 min, then 20 cycles of denaturation at 95 °C for 20 s, annealing at 56 °C for 30 s and extension at 72 °C for 4 min, followed by a final extension step at 72 °C for 5 min. PCR assembly products were extracted from the emulsion using isobutanol (2-Methyl-1-propanol, Sigma) and the Zymo DNA Clean & Concentrator-5 kit (Zymo Research) as previously published [20]. The resulting DNAs were loaded on a 1% TBE-agarose gel and separated with 100 V for 45 min. Assembly products of 700–800 bp were size-selected from the agarose gel, recovered using a Zymoclean Gel DNA Recovery kit, eluted in 6 mM Tris-Cl pH 8, and stored at − 20 °C until further analysis.

### Nested PCR amplification of human B cell assembly products

For specific further amplification of the HC-LC assembly products, a nested PCR amplification was performed with nested primers specific for IgM, IgG, and IGK constant regions (Additional file 1: Table S2). The PCR reaction contained nested primers at 0.4 μM concentrations, 200 μM dNTP mix, 1× Q5 reaction buffer and 0.02 U/μL Q5 high-fidelity DNA Polymerase (New England Biolabs) in a reaction volume of 50 μL with 3 μL of assembled DNA. Nested PCR amplification was performed with the following thermocycling conditions: initial denaturation at 98 °C for 3 min, then 34 cycles of denaturation at 98 °C for 30 s and annealing/extension at 71 °C for 1 min, followed by a final extension step at 72 °C for 5 min. Samples were collected after three different PCR cycle numbers (28, 31, and 34 cycles). Amplified PCR products were loaded on 1% TBE-agarose gels and separated with 100 V for 60 min. The desired products of ~ 710 bp were extracted as described above, sequencing libraries were prepared following the Illumina TruSeq DNA sample preparation guide and 2 × 250 base paired-end reads were sequenced using the Illumina MiSeq platform.

### Bioinformatic analysis of paired antibody heavy and light chain repertoires

Demultiplexing of 2 × 250 base paired-end reads from the MiSeq sequencing platform was performed based on adapter indices and sequencing data were obtained in fastq format. Only reads with minimum Phred quality scores of 10 over 50% of all nucleotides were retained and scanned for IgM, IgG, and IgK constant region sequences. Read pairs lacking constant region sequences or showing HC-HC or LC-LC structure were filtered out and the remaining reads were converted into fastq format and used as input for analysis with MiXCR (v1.2) [21] for alignment of reads to reference V(D)J and C gene sequences from the IMGT database [22], extraction, and clustering of CDR-H3 nucleotide (Additional file 1: Table S1). HC-CDR3 sequences containing frameshifts or stop codons and with less than two reads were filtered out. We created a HC-LC pairing statistics file to demonstrate paired VH-VK gene usage in the total paired HC-LC gene repertoires. Heat maps were generated using R and graphically displayed using ggplot2. Next, inter-individual TT-specific HC-CDR3 sequences were identified by comparing HC-CDR3 amino acid sequences obtained from the post-Td booster immunization sample to previously reported TT-specific HC-CDR3 sequences [23–26].

### PCR amplification of full-length HC and LC sequences

We designed a two-step PCR-based amplification method (Additional file 1: Figure S5) to incorporate restriction digestion sites to potentially TT-specific HC and LC with complete V(D)J gene sequence. This enabled efficient cloning of HC and LC sequences into respective expression vectors as well as production of recombinant antibodies for in vitro binding studies. Briefly, we selected 14 paired HC-LC CDR3 clonotypes obtained from IgG sequencing post-Td booster immunization based on their frequency, pairing accuracy, and fold difference between top1 LC-CDR3 and top2 LC-CDR3 paired to the given HC-CDR3 sequence. We extracted total RNA from frozen B cells isolated from post-Td booster immunization using TRIzol reagent (Ambion) purification according to the manufacturer’s instructions. In the first step, RT-PCR amplification for each selected HC- and LC-CDR3 clonotype was performed separately using the dART 1-step RT-PCR kit (Roboklon). The RT-PCR master mix (25 μL) contained HC and LC V gene-specific forward primers with BssHII restriction site overhangs together with individual CDR3-specific reverse primers with 18 nucleotides of FR4 region at 0.4 μM concentrations (Additional file 1: Table S3 and Figure S5), 1× dART 1-step RT-PCR master buffer mix, 1× dART master enzyme mix, and 4.5 ng total RNA. Thermocycling conditions were as follows: reverse transcription at 55 °C for 30 min, initial denaturation at 95 °C for 3 min, then 23 cycles of denaturation at 95 °C for 20 s, annealing at 56 °C for 30 s and extension at 72 °C for 90 s, followed by a final extension step at 72 °C for 5 min. RT-PCR products were purified using the Agencourt AMPure XP – PCR purification kit (Beckman Coulter) following the manufacturer’s instructions and eluted in 6 mM Tris-Cl pH 8.0. In the second step, purified RT-PCR products were used as template for PCR amplification using Q5 high-fidelity DNA polymerase (New England Biolabs). The nested PCR master mix (50 μL) contained forward primers encoding a BssHII restriction site and three nucleotides of HC or LC germline gene sequence together with reverse primers containing the complete FR4 region and NheI/HindIII restriction overhangs at 0.4-μM concentrations (Additional file 1: Table S3 and Figure S5), 4 μL of purified DNA, 200 μM dNTP mix, 1× Q5 reaction buffer, and 0.02 U/μL Q5 high-fidelity DNA Polymerase (New England Biolabs). Thermocycling conditions were as follows: initial denaturation at 98 °C for 3 min, then 16 cycles of denaturation at 98 °C for 30 s, annealing at 69 °C for 30 s and extension at 72 °C for 1 min, followed by a final extension step at 72 °C for 5 min. PCR products were separated on a TBE-agarose gel, full-length HC and LC amplicons with restriction digestion sites were extracted from the gel using the Zymoclean Gel DNA Recovery Kit (Zymo Research) and products were stored at − 20 °C until further use.

### Cloning and expression of recombinant monoclonal antibodies

Restriction digestion of full length HC and LC inserts and expression vectors (pCMV-CD30-4IE3_HC and pCMV-CD30-4IE3_LC) was performed with the restriction enzymes BssHII, NheI and HindIII (New England Biolabs). The resulting products were loaded on 2% TBE-agarose gels and bands of ~ 5.9 kb for the HC vector backbone, 5.3 kb for the LC vector backbone, ~ 370 bp for HC inserts, and ~ 340 bp for LC inserts were size-selected on agarose gels and purified as described above. Ligations of the corresponding inserts and vectors for the amplified HC and LC clonotypes were performed using instant sticky-end DNA ligase (New England Biolabs) and transformed into one-shot chemically competent *E. coli* TOP10 cells (IBA) following the manufacturer’s instructions. Plasmid DNAs were isolated from transformed colonies (8–16 colonies) using the QIAprep spin miniprep kit (Qiagen); similarities to the consensus sequences were confirmed using capillary Sanger sequencing. HC and LC plasmid DNA sequences that matched closest to the consensus sequences were co-transfected into human embryonic kidney cell line HEK 293 T (ATCC, CRL-11268) cells. HEK 293 T cells were cultured using rich glucose (4.5 g/L D-glucose) Dulbecco’s Modified Eagle’s Medium (Gibco BRL) supplemented with heat-inactivated ultra-low IgG fetal bovine serum (Thermo Fisher Scientific), 100 U/mL penicillin, and 100 μg/mL streptomycin. Purified plasmid DNAs for paired HC and LC clonotypes were co-transfected into 85–95% confluent HEK 293 T cells using PEI (polyethyleneamine, Polysciences). Culture supernatants were collected four days after transfection and TT antigen-specific clonotypes were identified by indirect ELISA.

### Enzyme-linked immunosorbent assays (ELISA)

We performed indirect ELISA assays to identify mAbs derived from the immunized proband binding to TT antigen using the transfected cell culture supernatants. Nunc-Immuno MicroWell 96-well solid plates (Thermo Fisher Scientific) were coated with 100 μL of 10 μg/mL TT antigen (Statens Serum Institute, Copenhagen, Denmark) in 50 mM carbonate buffer pH 9.6, incubated overnight at 4 °C, washed three times with PBS, and blocked with 2% non-fat dried milk (Bio-Rad) in PBS for 150 min at room temperature. After blocking, 120 μL of 1:2 serially diluted transfected supernatants in PTM (PBS, 0.1% Tween-20, 2% non-fat dried milk) were added to the wells, 350 ng of mouse anti-TT mAb (GeneTex) was applied to one well as a positive control, and plates were incubated for 1 h at room temperature. Plates were washed three times with PBS-T (0.1% Tween-20) and 50 μL of a 1:2000 dilution of goat anti-human kappa LC-HRP secondary antibody (Thermo Fisher Scientific) were added to the wells, 50 μL of a 1:2000 dilution of goat anti-mouse IgG HC-HRP secondary antibody (Sigma #A0168) were added to the positive control well, plates were incubated for 2 min at room temperature and washed three times with PBS-T. For color development, we added 50 μL of one-step Ultra TMB-ELISA substrate (Thermo Fisher Scientific) per well, incubated the plates for 5 min at room temperature, and stopped the Ag:Ab binding reaction by addition of 50 μL 2 M H_2_SO_4_. The absorbance was measured at 450 nm using the GloMax Multi Detection System (Promega). ELISA assays for all clonotypes were performed in triplicates, the values were normalized to remove background signals, and errors were represented as standard deviations from the mean.

### Analysis of chimeric amplicon formation during nested PCR

Four defined HC-LC amplicons were generated by amplifying the HC and LC from the respective pCMV plasmids (see above) and using a PCR assembly reaction to generate the four distinct HC-LC assemblies. HC and LC plasmid DNAs were used as templates for PCR amplification of the Top1, Top2, Top3, and Top4 clonal chain pairs using primers specific for the respective VH and VK gene families and IgG and IgK constant regions (Additional file 1: Table S2 and Figure S6a). Purified plasmid DNA (10 ng) was added to each 25 μL PCR reaction containing 0.4 μM of each primer, 200 μM dNTP mix, 1× Q5 reaction buffer, and 0.2 U/μL Q5 high-fidelity DNA Polymerase. Thermal cycling was performed with initial denaturation at 98 °C for 3 min, followed by 25 cycles of denaturation at 98 °C for 30 s, annealing/extension at 71 °C for 1 min (for HC plasmid DNA) or annealing at 64 °C for 1 min and extension at 72 °C for 1 min (for LC plasmid DNA), followed by a final extension step at 72 °C for 5 min. PCR products were loaded onto separate 1% TBE-agarose gels and separated with 100 V for 60 min. The desired DNA products of ~ 400 bp (for HC) and ~ 350 bp (for LC) were size-selected and extracted from the gel as described above. The purified HC and LC PCR products were used as templates for HC and LC assembly by overlap extension PCR (Additional file 1: Figure S6b). Briefly, 5 ng of each HC and corresponding paired LC DNA were added into each 50 μL PCR reaction containing 1× dART 1-step RT-PCR master buffer (Roboklon), 2× dART master enzyme (Roboklon), and 0.4 μM of each IgG and IgK constant region primer (Additional file 1: Table S2). Thermal cycling was performed with RT inactivation at 95 °C for 3 min, followed by three cycles of denaturation at 95 °C for 20 s, annealing at 56 °C for 30 s and extension at 72 °C for 2 min, followed by 25 cycles of denaturation at 95 °C for 20 s, annealing at 56 °C for 30 s and extension at 72 °C for 4 min, followed by a final extension step at 72 °C for 5 min. The assembly products were loaded onto 1% TBE-agarose gels and separated with 100 V for 45 min. The assembly products of ~ 750 bp were size-selected and extracted from the agarose gel as described above. The individually assembled HC-LC clonal pairs were pooled together and used as template for nested PCR amplification with primers specific for IgG and IgK constant regions (Additional file 1: Table S2, Figure S6c). The nested PCR reaction and thermal cycling conditions were the same as described in the “Nested PCR amplification of human B cell assembly products” section except that the PCR amplification was performed for 25 cycles. PCR products were loaded onto 1% TBE-agarose gels, separated with 100 V for 60 min and desired products of ~ 720 bp were extracted as described above. Sequencing libraries from the individual assemblies and from the mixed assemblies after nested PCR were prepared following the Illumina TruSeq DNA sample preparation guide and 2 × 250 base paired-end reads were generated using the Illumina MiSeq platform.

## Results

### Microsome-associated mRNAs can be used to retain native antibody HC-LC pairs with high pairing accuracy

**Fig. 1:**
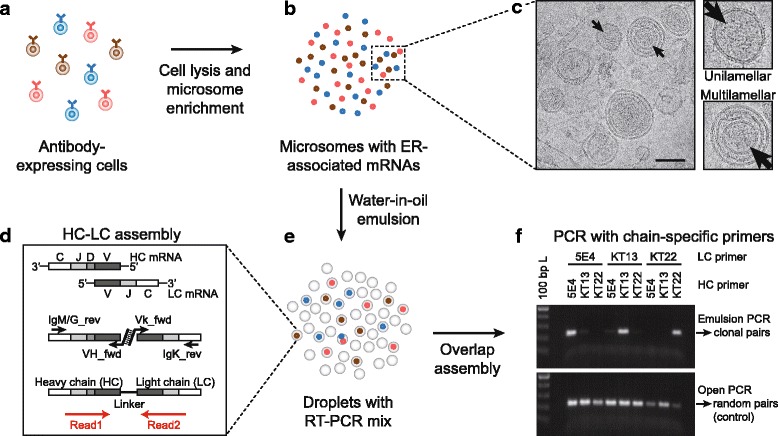
Overview of paired antibody HC-LC amplification using microsomes in water-in-oil emulsion droplets. **a** Antibody-expressing cell populations were used for microsome preparation. **b** Cells were lysed using a sucrose buffer with 5% digitonin and microsomes with rER-associated mRNAs were enriched using differential centrifugation. **c** Transmission electron microscopy showed enriched rER microsomes with multilamellar and unilamellar structures. The image was acquired from HEK 293T microsomes used for establishment of the method. Scale bar represents 100 nm. **d** HC and LC mRNAs were assembled by overlap extension RT-PCR to generate natively paired HC-LC amplicons using constant region primers for reverse transcription and variable region primers for overlap extension assembly. The location and orientation of the paired-end MiSeq reads on the amplicons are indicated by *red arrows*. **e** The assembly reaction was carried out within individual emulsion droplets with microsomes from single cells for clonal assembly of rER-associated mRNAs. **f** Nested PCR amplification with hybridoma-specific nested primers on the assembled DNA demonstrated strong enrichment of native HC-LC pairs when using emulsion PCR during the assembly reaction (*upper panel*), while a control showed random pairing of heavy and light chains when using conventional open PCR during the assembly reaction

Our approach is based on the concept that each B cell contains rough endoplasmic reticulum (rER) with bound ribosomes for co-transcriptional translocation of secretory proteins. These bound ribosomes are thus associated with both Ig HC and LC mRNAs, located at translocon complexes [27], which are translated into membrane-bound or secretory antibodies. We reasoned that rER microsomes obtained after cell lysis should retain the correctly paired HC and LC mRNAs of each individual B cell and thus represent the smallest sub-cellular entity comprising both types of mRNAs. It is likely that several microsomes are generated from each cell which leads to a higher clonal redundancy for a more efficient library synthesis when compared to using whole cells as templates. Therefore, these microsomes can subsequently be used for clonal RT-PCR assembly of the two chains from the original single cells, providing that the derived microsomes are separated into individual reaction vessels, a step that we have carried out by using water-in-oil emulsions. The entire workflow is summarized in Fig. 1.

We developed our method using HEK 293T cells based on a protocol for preparation of microsomes from plant material [28]. To preserve the mRNAs at rER translocon complexes, we first treated the cells with the protein synthesis inhibitor cycloheximide [29] to retain stalled ribosomes with associated mRNAs in the resulting microsomes. The cycloheximide-treated cells were incubated in a sucrose buffer containing 5% digitonin, leading to the lysis of cells and organelles during which rER sheets collapse and form multilayered structures preserving the mRNA transcripts while keeping the cell nuclei intact. The sucrose provides higher density inside the lysed microsomes. Then, cell debris, nuclei, non-secretory mRNAs, and mitochondria were removed by low-speed centrifugation (600 g). This purification step has the advantage to greatly reduce PCR artefacts due to off-target amplification by mispriming on genomic DNA and other mRNAs. The microsome-containing supernatant was diluted with water so that microsomes could subsequently be pelleted based on their higher buoyant density using high-speed centrifugation (20,800 g) in a cooled table-top centrifuge. After removal of the supernatant (cytosol), the microsomes were resuspended in wash buffer and sedimented again (20,800 g) to further enrich microsomes for downstream applications (Fig. 1b). For verification of our microsome preparation method, enriched rER-microsomes from HEK 293T cells were visualized using transmission electron microscopy (Fig. 1c). We observed that the majority of microsomes were composed of multi-lamellar vesicles of approximately spherical shape, while some others were of uni-lamellar structure. This result suggested that our method can be used to obtain stable rER microsomes, thus avoiding the use of tedious ultracentrifugation steps [28].

Next, we tested if the enriched rER microsomes could be used for clonal assembly and amplification of paired immunoglobulin HC-LC from single cells. For this, we mixed cells from three mouse hybridoma cell lines with known Ig HC and LC sequences (cell lines 5E4, KT13, and KT22) and prepared microsomes from the cell mix according to our protocol (Fig. 1a and b, Additional file 1: Figure S1). We then passed the microsomes into water-in-oil emulsion droplets containing RT-PCR assembly master mix with overlap extension primers (Fig. 1d), wherein, based on Poisson statistics, the vast majority of individual microsomes were encapsulated in separate emulsion droplets (Fig. 1e). If clonal pairing and amplification occur, the amplified sequences should be strongly enriched for the three correct chain pairs among the nine possible pairings of three different HCs and LCs (Additional file 1: Figure S1). Within the emulsion droplets, the HC and LC mRNAs from individual microsomes were reverse transcribed using isotype-specific primers (IgM and IgK), assembled by overlap extension PCR and amplified. After size selection of the assembled DNA on an agarose gel, subsequent nested PCR with hybridoma-specific primers showed that the three correct chain pairs were strongly enriched (> 95%) versus the nine possible permutations (Fig. 1f, upper panel, and Additional file 1: Figure S1). In contrast, we observed no enrichment of the correctly paired chains in control experiments performed in parallel where the assembly was carried out in conventional open PCR without emulsification, leading to an evenly balanced random chain assembly (Fig. 1f, lower panel). We quantified the amount of cross-contamination in the assembled DNA using real-time quantitative PCR (Additional file 1: Figure S1f) and found that cross-contamination among the distinct hybridomas was present at 0.2% frequency, while 99.8% of the chains demonstrated correct pairing. These results show that our method is suitable for clonal amplification of paired Ig HC and LC from single cells with high pairing accuracy.

### A scalable high-throughput sequencing platform to retain native antibody HC-LC pairs from single B cells

**Fig. 2:**
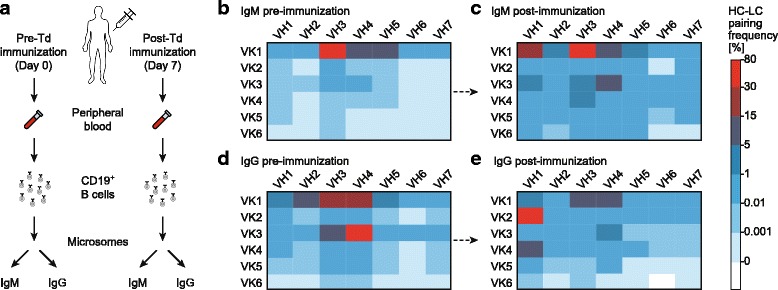
Gene usage of different immunoglobulin VH and VK gene families observed in paired HC-LC gene repertoires before and after Td booster immunization. **a** CD19^+^ B cells were isolated from freshly derived peripheral whole blood from a healthy proband before (day 0) and after (day 7) Td booster immunization. Amplification of paired antibody HC-LC repertoires was performed as described in Fig. 1 and paired libraries were sequenced on the Illumina MiSeq platform. Each *panel* represents sequencing data obtained from one independent emulsion RT-PCR assembly reaction. **b** IgM HC-LC pairing frequencies from pre-immunization CD19^+^ B cell sample (total aligned reads: 5,238,212; final clonotype count: 2200). **c** IgM HC-LC pairing frequencies from seven days post-Td booster immunization (total aligned reads: 4,647,787; final clonotype count: 4031). **d** IgG HC-LC pairing frequencies from pre-immunization CD19^+^ B cell sample spiked with 0.5% ARH-77 microsomes (total aligned reads: 4,411,684; final clonotype count: 4841). **e** IgG HC-LC pairing frequencies from seven days post-Td booster immunization CD19^+^ B cell sample spiked with 0.5% ARH-77 microsomes (total aligned reads: 4,332,934; final clonotype count: 2872). *Colors* indicate percentage of reads for indicated VH-VK pairings among all reads in the analyzed B cell repertoire

We then applied our method to study immunization-induced changes in CD19^+^ B cell repertoires from pre- (day 0) and post- (day 7) Td booster immunization (Fig. 2). We used 1.5 million CD19^+^ B cells freshly isolated from peripheral whole blood samples of a healthy donor both pre- and post-Td booster immunization and prepared microsomes enriched with rER. As a control for native HC-LC pairing, we prepared microsomes from frozen ARH-77 cells expressing known IgG HC and IgK LC sequences and spiked 0.5% (v/v) of ARH-77 microsomes into B cell-derived microsomes (Additional file 1: Table S1). The microsomes were passed into water-in-oil emulsion droplets for amplification in two separate reactions with primers specific for IgM and IgG isotypes, respectively. After emulsification, overlap-extension RT-PCR, and nested PCR (Additional file 1: Figure S2), we prepared Illumina TruSeq libraries from the nested PCR amplicons and performed sequencing on the Illumina MiSeq with paired reads of 2 × 250 bases (Additional file 2: Figure S3). The raw sequencing reads were quality filtered and annotated to define the individual HC (IgM or IgG) and LC (IgK) isotypes. The annotated reads were aligned to the human Ig germline genes (IMGT annotation [22]) and clustered using MiXCR [21] to determine the number of unique paired CDR3 clones (requiring ≥ 2 reads per pair) including correction of PCR errors. From the pre-immunization sample, we identified a total of 2200 and 4841 HC-LC pairs for IgM and IgG, respectively (Additional file 2: Data files S1 and S2). The post-Td immunization sample resulted in 4031 and 2872 HC-LC pairs for IgM and IgG, respectively (Additional file 2: Data files S3 and S4). Among these, we identified 212 (IgM) and 125 (IgG) HC-CDR3 clonotypes that were present in both the pre- and post-Td immunization samples. Of these, 50.0% (IgM) and 60.0% (IgG) of the HC-CDR3s found in pre- and post-Td booster immunization data shared the same LC-CDR3 sequences, demonstrating the application of this technology to identify and track pre-existing B cells, possibly from the antigen-specific memory B cell compartment [30] (Additional file 2: Data files S5 and S6). The ARH-77 spike-in HC-LC pairing demonstrated preferential pairing of the known HC with the correct corresponding LC (Additional file 1: Figure S4). Of the IgM and IgG isotypes pre- and post-Td immunization, the top ten pairs constituted 57% and 49% (for IgM isotype) and 61% and 76% (for IgG isotype) of the total aligned reads, respectively, indicating a clonotype distribution that is skewed towards the most frequent HC-LC pairs.

We then generated heat maps showing the HC-LC pairing frequencies of all aligned reads and observed strong changes in VH gene family usage and expansion of certain B cell clones in response to antigen stimulation [2, 23, 30, 31]. Specifically, we found that certain VH-VK pairings (e.g. VH3-VK1, VH4-VK1, and VH4-VK3) were highly frequent (up to 78% of total reads) in the pre-immunization samples for both the IgM and IgG isotypes (Fig. 2b and d). Post Td booster immunization, other pairings such as VH1-VK1, VH1-VK2, VH3-VK1, and VH4-VK1 were predominantly observed in both IgM (Fig. 2c) and IgG (Fig. 2e) isotypes. We also identified rare HC-LC pairs (e.g. VH7-VK5 and VH7-VK6) that are generally observed at lower frequencies (Fig. 2b–e), as reported in prior studies [10, 32]. This result illustrates the sensitivity of our technique to identify rare clonal pairs.

We quantified the presence and frequency of promiscuous LC sequences (LC paired to more than one specific HC) among all identified HC-LC pairs in all four samples (Additional file 3: S1-S4). We observed that three samples (IgG pre- and post- immunization, and IgM pre-immunization) contained 15–17% promiscuous LC, while one sample (IgM post-immunization) showed a higher frequency of 38.7% promiscuous LC. These observations are in line with previous studies reporting LC promiscuity due to lower theoretical diversity of LC junctions [15, 33]. We further compared the IgG HC-CDR3 amino acid sequences obtained from post-Td booster immunization with TT-specific HC-CDR3 sequences from previous studies [24–26, 30]. We found two previously reported TT antigen-specific HC-CDR3 sequences in our dataset (CARQADNWFDPW and CATGRTLDYW) [24, 30], suggesting the suitability of our method to track known sequences related to diseases and autoreactive antibodies [2].

### Application of paired antibody HC-LC repertoire sequencing for antigen-specific mAb discovery

**Fig. 3:**
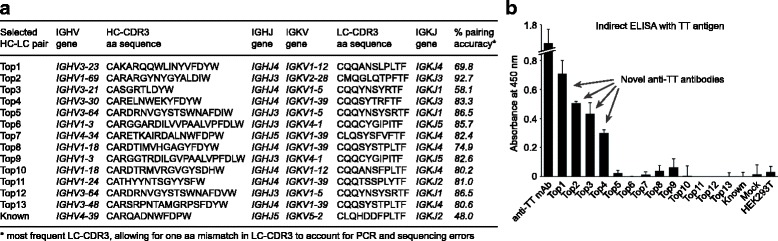
Binding studies of selected IgG antibodies induced in the post-Td booster immunization sample. **a** Fourteen highly induced HC-LC pairs including one known TT antigen-specific HC-CDR3 sequence were cloned into IgG HC and LC expression vectors, co-transfected, and expressed in HEK 293T cells for production of IgG mAbs. **b** Indirect ELISA using TT antigen and transfected HEK 293T cell supernatants reveals four novel anti-TT antibodies (named Top1, Top2, Top3, and Top4). A commercial TT-specific mAb used as positive control in the assays (anti-TT mAb) resulted in a strong signal while the negative controls (Mock – Mock transfection; HEK 293T – Cell culture supernatant from untransfected cells) resulted in low background signals

Finally, we demonstrated that our paired sequencing technique is suitable for the discovery of novel antigen-specific human monoclonal antibodies (mAbs) by performing antibody cloning, expression and antigen binding studies using ELISA. We selected 14 highly induced HC-LC pairs from the IgG B cell repertoire post-Td booster immunization, including the HC-LC pair for one previously reported TT-specific HC-CDR3 sequence (CARQADNWFDPW) (Fig. 3a). We used a two-step PCR strategy for incorporating restriction digestion sites to the selected HC-LC pairs (Additional file 1: Figure S5) for cloning into IgG HC and LC expression vectors. For recombinant mAb production, the HC and corresponding LC plasmids were co-transfected into HEK 293T cells (Additional file 1: Figure S5) and IgG-containing cell culture supernatants were harvested on day 4 after transfection. We performed indirect ELISA experiments with the transfected cell supernatants using plates coated with TT antigen and identified four novel TT-specific mAbs, named Top1, Top2, Top3, and Top4 here (Fig. 3b). Interestingly, the Top2, Top3, and Top4 HC-LC pairings were also present in the sequenced pre-immunization repertoire, albeit at much lower frequencies (< 0.1% of total reads), suggesting the clonal expansion of pre-existing clonotypes after antigen exposure [30]. However, the previously reported TT-specific HC-CDR3 clonotype CARQADNWFDPW did not bind to TT antigen using our experimental setup (Fig. 3b), probably because it is a so-called public rearrangement with limited introduction of N/P nucleotides that is associated to IGHV4–39 and IGKV5–2 in our study, while similar TT-specific binders with this type of CDR3 are associated to IGHV4–30-2 and IGKV3–15. Also, the paired LC-CDR3 was different in our study (CLQHDDFPLTF) compared to the LC-CDR3 previously identified from a TT-binding memory B cell (CQQYYNWPPYTF) [26]. Our results show that almost one-third (29%) of the selected antibodies identified by our method did bind to TT antigen, thus demonstrating the applicability of this method for rapid discovery of mAbs using native Ig chain pairing information from B cells.

Immune repertoire sequencing methods can be affected by the formation of chimeric amplicons during PCR amplification [34]. To address this potential issue and to quantify the amount of chimeric amplicons generated during the second (non-emulsion) stage of our method, we generated and mixed four defined clonal HC-LC amplicons (from the Top1, Top2, Top3, and Top4 antibodies), performed the secondary PCR step and sequenced the resulting amplicons on the MiSeq platform with 2 × 250 bases (Additional file 1: Figure S6a–c). In parallel, we also sequenced the initial clonal amplicons individually as control for amplicon purity before secondary PCR. The reads generated from the initial amplicons before PCR showed > 99.6% correct HC-LC pairs (Additional file 1: Figure S6d), with < 0.4% chimeras that were probably generated during bridge amplification for cluster generation on the MiSeq flow cell. Analysis of the reads from the mixed amplicons after secondary PCR showed that the PCR step indeed generated chimeric amplicons, with the extent of chimer formation depending on the sequence identity among amplicons (Additional file 1: Figure S6e). The three amplicons from the Top1, Top3, and Top4 antibodies, sharing HC V genes from the same IGHV3 superfamily, showed 10–14% chimera formation among each other, while the Top2 amplicon with IGHV1 superfamily V gene formed < 0.3% chimeras with the other three amplicons. The average amount of chimeric amplicon formation was 18.3% for the four amplicons tested here.

## Discussion

We developed a simple, cost-effective, and innovative approach for high-throughput sequencing of native antibody HC-LC pairs from B cell populations. In contrast to current paired Ig repertoire sequencing technologies [9–12, 15, 35], this method does not require the physical separation of single B cells using a flow cytometer, the construction of a flow focusing apparatus, or complex microfluidic devices. Our simple method relies on preparation of rER microsomes from B cells using a table-top centrifuge, avoiding ultracentrifugation steps [28]. The use of rER microsomes to link native HC-LC pairs in emulsion droplets overcomes previously reported difficulties involving cell entrapment in emulsion droplets, cell lysis, and RNA degradation during PCR [35, 36]. Due to the removal of the bulk genomic DNA and non-secretory RNA during microsome preparation, PCR artefacts due to off-target amplification by mispriming are greatly reduced. The simplicity of this method makes it widely applicable, also for laboratories without specialized equipment.

We report that our method can efficiently capture thousands of antibody HC-LC clonal pairs (Additional file 2: Data files S1–S4) by processing over one million B cells per experiment. Our observation that the top ten HC-LC sequence pairs accounted for 49–76% of the total aligned reads indicated a skewed clonotype distribution in the sequenced repertoires. An explanation for this is that our method predominantly detects antibody mRNAs that are present in higher abundance in the analyzed B cell population. This is because this method does not use single intact B cells, but rather ER microsomes derived from B cells, for antibody chain assembly. Cells with larger secretory ER volumes, which are secreting high amounts of antibodies, contribute a larger fraction of antibody sequences to the resulting dataset. Therefore, we expect that our data does not reflect actual B cell frequencies, but instead reflects the amounts of secreted antibody molecules. Thus, the thousands of heavy-light chain pairs we detected from over one million B cells represent the subset of cells with the highest antibody production (e.g. plasma cells), which is actually a very interesting cell subset when looking for antigen-specific antibodies. Also, we noticed preferential amplification of certain V-gene segments [8, 37], reflecting amplification biases in favor of the primers used for the VK1 and VK3 LC gene families (Fig. 2b–e), and thus the reported HC-LC pairs inadequately represent the actual clonal frequencies. However, a more accurate estimate of the human B cell repertoire using our method is possible through integrating relatively simple optimizations such as minimization of PCR primer biases by adjusting primer concentrations, limiting the amplification cycles as well as by the use of unique molecular identifiers (UMI) to reduce sequence-dependent amplification biases in the nested PCR amplification [37, 38].

We observed that the IgM repertoires obtained from CD19^+^ B cells demonstrated relatively low clonal diversity in the pre-immunization sample in comparison to the post-immunization sample. In contrast, the IgG clonal diversity in the post-immunization sample is lower than in the pre-immunization sample, indicating that post-immunization, the IgG repertoire was dominated by antigen-experienced clonal pairs.

We showed that our paired antibody sequencing method was adequately sensitive in detecting antigen-specific B cell clones occurring at lower frequencies. This was demonstrated by the identification of three out of four novel TT-specific antibody sequences that were also found at low frequencies in the IgG pre-immunization sample. Our method can therefore track the expansion of B cells from pre- to post-immunization [2, 24] for the discovery of antigen-specific mAbs [1–3, 10]. However, it must be noted that our method, as reported here, is dependent on highly expanded sequences post-immunization in order to identify novel antigen-specific sequences, thereby limiting the application of our method to identify antigen-specific sequences from pre-immunization samples. An improved strategy to determine antigen-specific antibody sequences from B cell repertoires before immunization would be to pre-sort or enrich B cells according to antigen specificity, so that the resulting paired antibody sequences are highly enriched for antigen-specific sequences [39].

Using a set of defined amplicons, we detected an average amount of 18.3% chimeric amplicon formation during the second (non-emulsion) stage of our method, which is below an extent that would prevent the applicability of this method for rapid discovery of mAbs. We expect that also our repertoire data from human B cells before and after immunization contains similar numbers of chimeric amplicons, which would account for the observation that the pairing accuracy is less than what would be expected from the initial experiments using mouse hybridomas. Interestingly, the mouse hybridoma sequences used for establishment of the method showed only ~ 0.2% chimeric amplicons, probably due to their very divergent V gene sequences from distant V gene superfamilies. A method-specific mitigation strategy for computational removal of chimera pairs could be based on the inclusion of short unique molecular identifier (UMI) sequences next to the overlap sequence for heavy chain assembly in the central part of the amplicons. These UMIs can be sequenced using an additional index read, which is possible on the MiSeq platform. The UMI sequences could then be used to computationally remove lower-frequency (chimeric) LC sequences for each specific combination of HC and UMI sequence, keeping only the most frequent truly paired light chains.

The whole process – from B cell isolation to sequencing paired Ig repertoires and analyzing HC-LC sequences – takes only four days. Antibody validation can be carried out within two weeks after sequencing data acquisition [10, 40]. Our approach can be combined with bioinformatic tools [41] or conventional screening technologies [42–45] to facilitate the rapid identification of antigen-specific mAbs thereby circumventing laborious large-scale screening of combinatorial B cell libraries [2, 45].

## Conclusions

The presented method provides a simple, cost-effective, and scalable platform to characterize native antibody HC-LC pairs at single-cell level for rapid identification and generation of antigen-specific monoclonal antibodies with minimal costs using only common laboratory equipment. Our simple method using rER-associated mRNAs to retain paired antibody HC-LC information from single cells can be widely applicable in labs that do not have commercially available specialized equipment. We anticipate that this technology could possibly accelerate translational research towards antibody discovery for diagnostics, therapeutics, cancer immunotherapies, and investigating immune responses to vaccination, cancer neoantigens, and various aspects of immune biology research.

## Additional files

Additional file 1: Contains supplementary **Figures. S1–S6** and **Tables. S1–S3**. (DOCX 10288 kb)

Additional file 2: Contains supplementary data files 1–6. (XLSX 1059 kb)

Additional file 3: Contains supplementary data files 1–4. (XLSX 71 kb)

## Abbreviations

CDR: Complementarity determining region
ELISA: Enzyme-linked immunosorbant assay
HC: Heavy chain
Ig: Immunoglobulin
IgG: Immunoglobulin G
IgK: Immunoglobulin Kappa
IgM: Immunoglobulin M
LC: Light chain
mAbs: Monoclonal antibodies
PCR: Polymerase chain reaction
qPCR: Quantitative polymerase chain reaction
rER: Rough endoplasmic reticulum
RT-PCR: Reverse transcription polymerase chain reaction
Td: Tetanus toxoid and Diphtheria toxoid
TT: Tetanus toxoid
UMI: Unique molecular identifier
V: Variable gene segment
VH: Variable heavy
VK: Variable kappa
