# Supplementary material for: Simple paired heavy- and light-chain antibody repertoire sequencing using endoplasmic reticulum microsomes
Source: Genome Med. 2018 Apr 27;10:34. doi: 10.1186/s13073-018-0542-5 (PMC5921987; doi:10.1186/s13073-018-0542-5)
Supplement: Supplementary file 1 — Contains supplementary Figures. S1–S6 and Tables. S1–S3. (DOCX 10288 kb) [file 13073_2018_542_MOESM1_ESM.docx]

**Additional file 1**


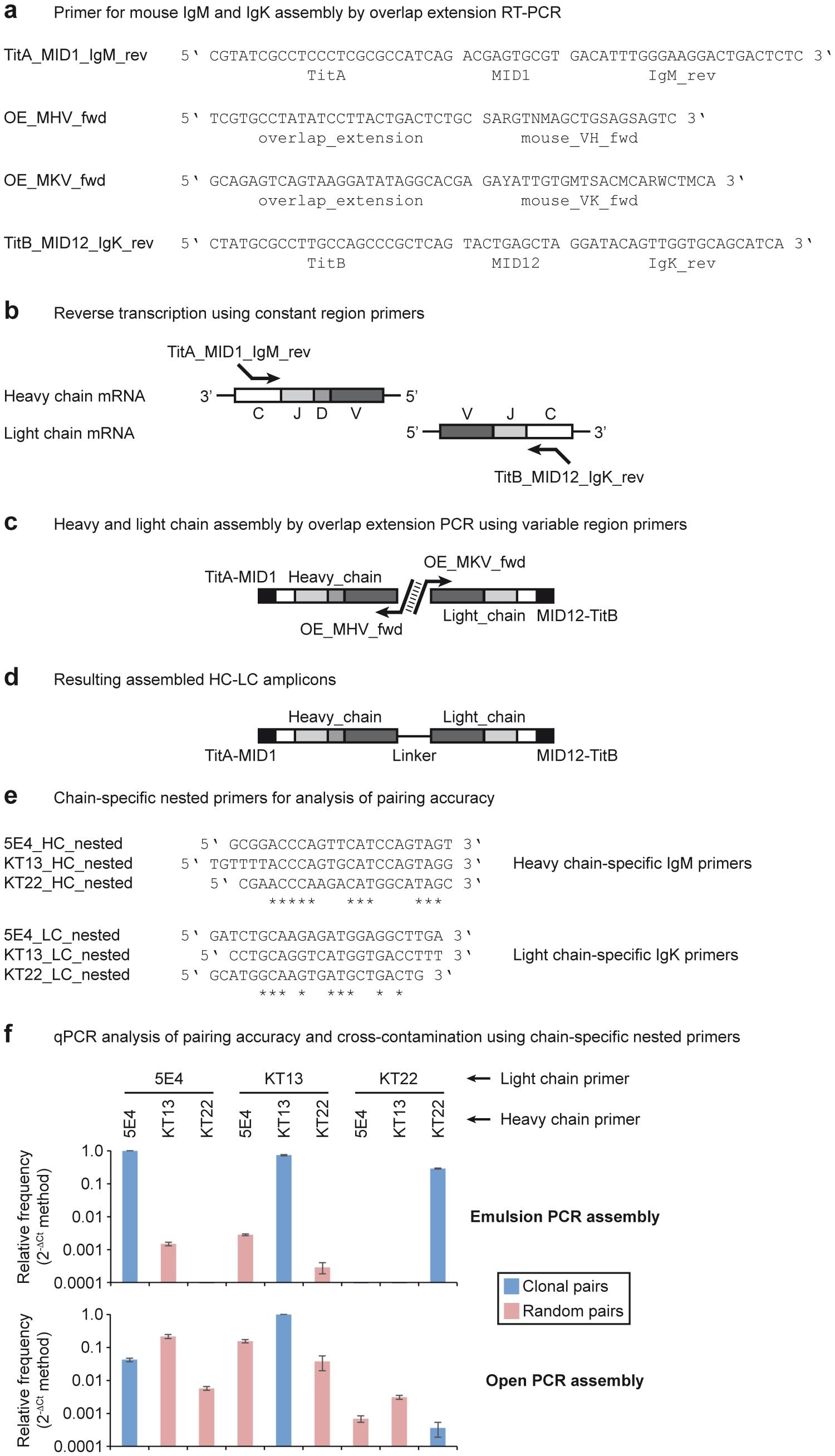


**Figure S1:** Overview of heavy and light chain assembly from mixed mouse hybridoma cell lines and analysis of pairing accuracy. (**a**) Primer sequences used for mouse IgM-IgK reverse transcription, assembly and amplification. Constant region primers included Roche 454 FLX Titanium adapters and multiplexing indices for high-throughput sequencing. Variable region primers included sequences for overlap extension by PCR assembly. The immunoglobulin-specific parts of the primers are based on the primers reported in Wang et al. J Immunol Methods 2000. (**b**) Reverse transcription from rER microsomes in emulsion was performed using IgM and IgK constant region primers including FLX Titanium adapters. (**c**) Heavy and light chain assembly was performed using variable region primers containing sequences for overlap extension. (**d**) The resulting heavy chain-light chain assembly products are flanked by FLX Titanium sequences and connected by the overlap extension linker sequence. (**e**) Chain-specific primer sequences used for analysis of heavy chain-light chain pairing accuracy by nested PCR. (**f**) Quantification of cross-contamination using real-time quantitative PCR on heavy-light chain assemblies from emulsion and open PCR using chain-specific primers. The assembly product from emulsion PCR (upper panel) showed 99.8% correct chain pairing of the hybridoma sequences, while the control assembly product from open PCR (lower panel) showed random mixing of chains.


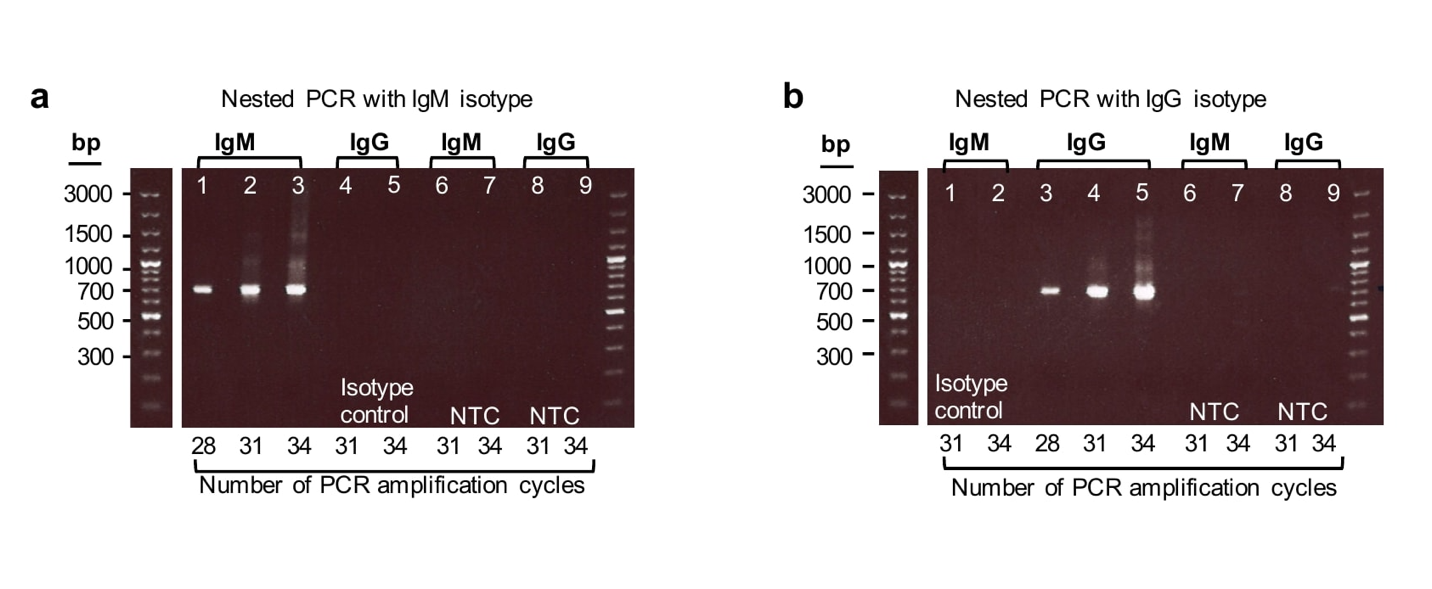


**Figure S2:** Amplification of nested PCR products with three different cycle numbers ranging from 28 to 34 cycles. (**a**) Nested PCR amplification of IgM-IgK amplicons from post-Td booster immunization samples. PCR amplification with IgM and IgK nested primers yields a single band at ~710 bp representing the assembled HC-LC DNA (lanes 1, 2 and 3). No background signals were observed in an isotype control (lanes 4 and 5) and a no-template control (NTC) with isotype-specific nested primers (lanes 6 to 9). (**b**) Nested PCR amplification of IgG-IgK amplicons from post-Td booster immunization samples.


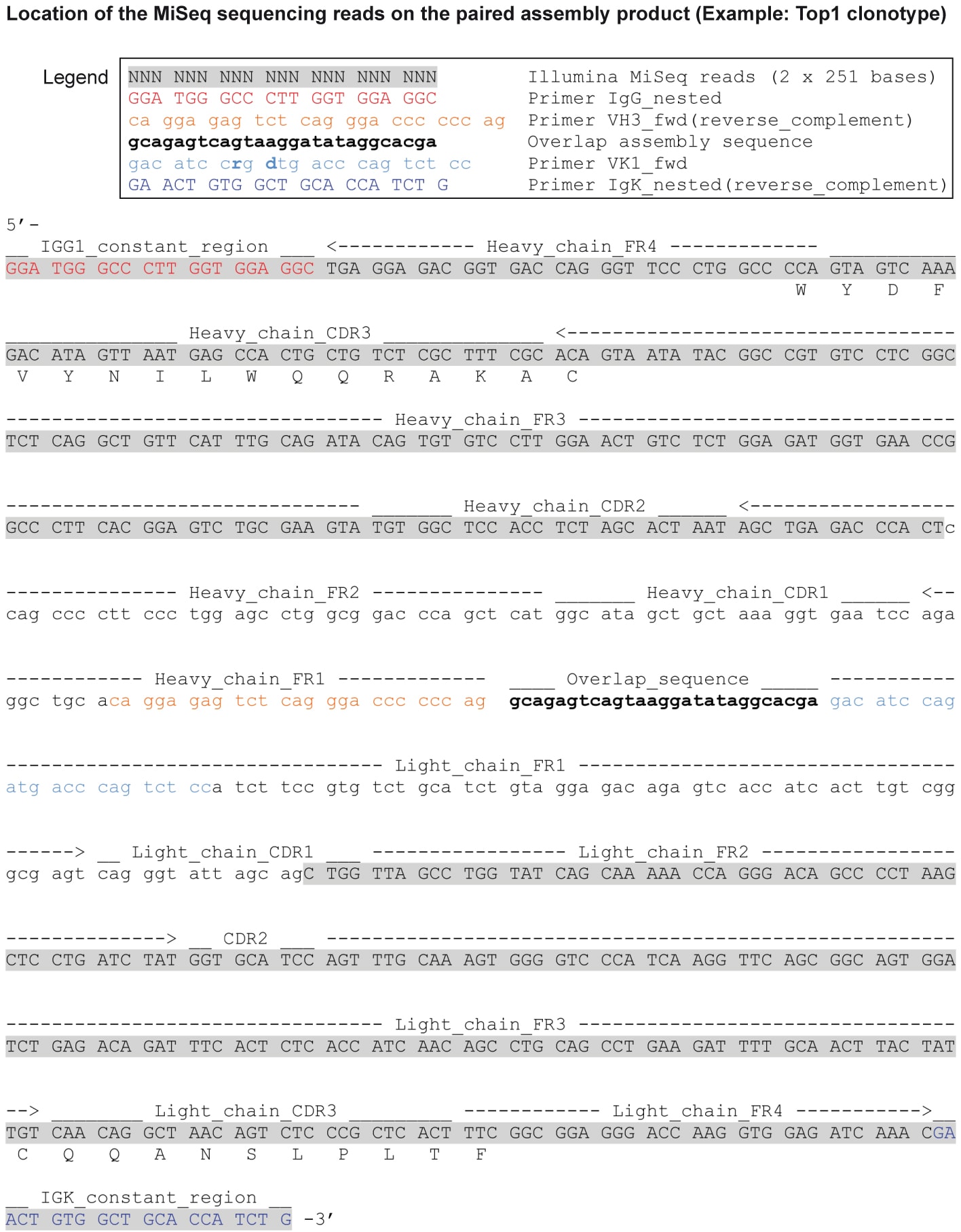


**Figure S3:** Schematic representation of the location of the MiSeq paired-end sequencing reads on an example assembly sequence (Top1) in the context of all framework regions (FR1-FR4), complementarity determining regions (CDR1-CDR3), constant regions (IgG and IgK) and the overlap sequence used for paired heavy and light chain assembly.


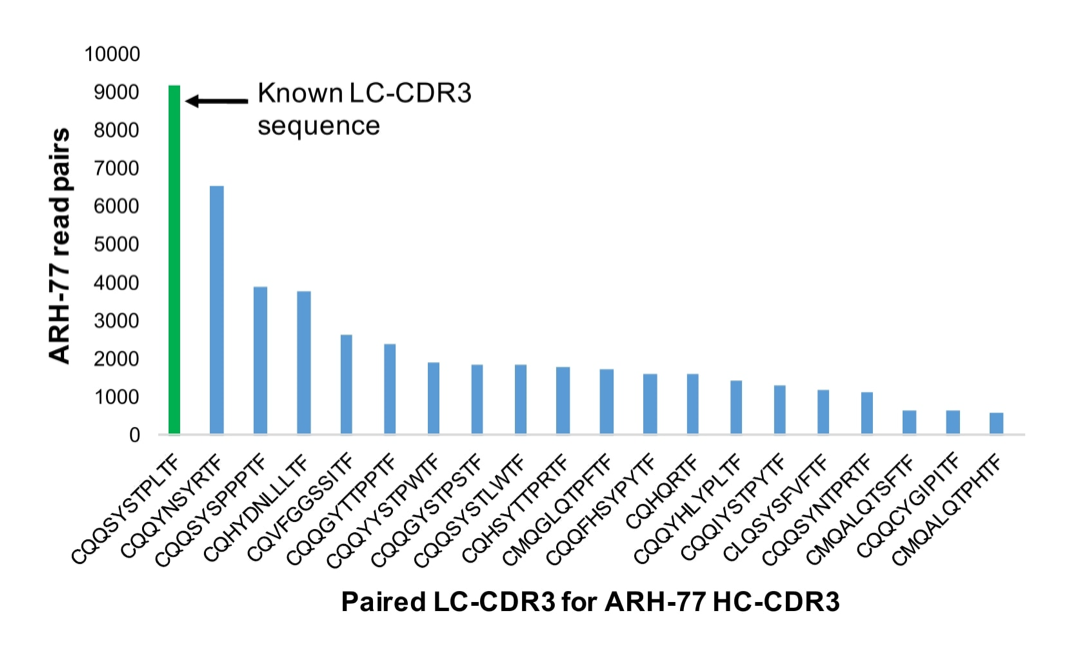


**Figure S4:** Light chain pairing distribution for ARH-77 cell spike-in post Td-booster immunization sample. The ARH-77 HC-CDR3 sequence was preferentially paired to the correct LC-CDR3 sequence (CQQSYSTPLTF, shown in green), with some mispairing to other light chains (shown in blue).


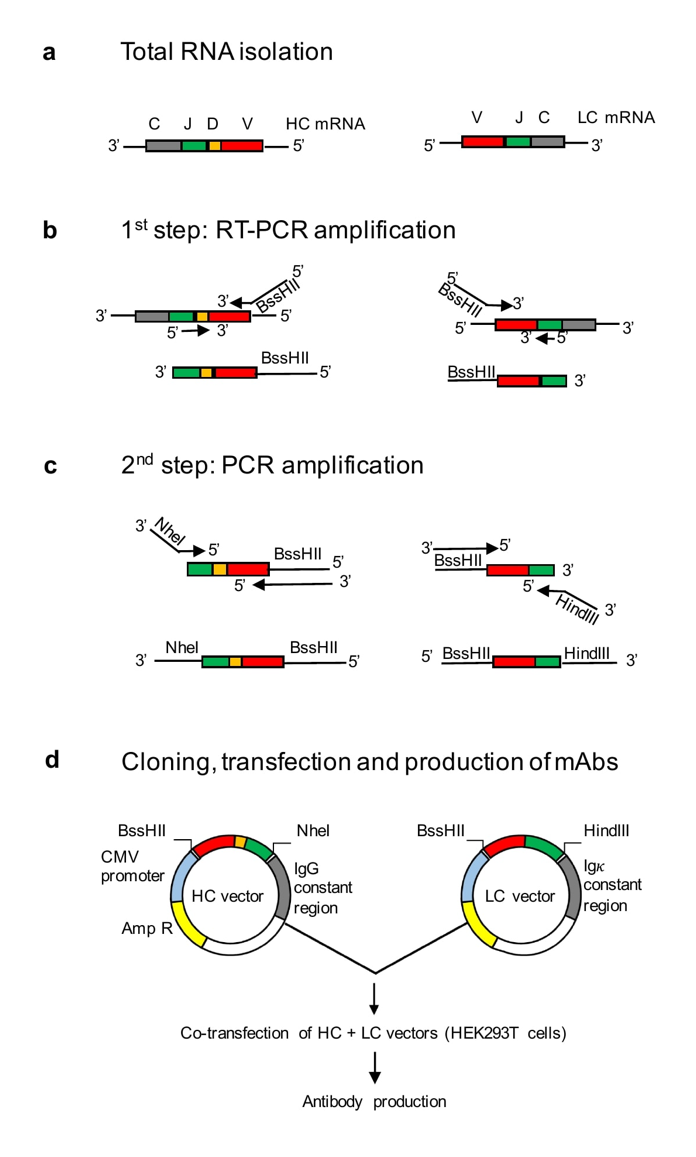


**Figure S5:** Schematic illustration of the procedure used for cloning antibody HC and LC into respective expression vectors for production of monoclonal IgG antibodies. (**a**) Total RNA was isolated from frozen B cells obtained after Td booster vaccination. (**b**) For selective amplification of individual clonotypes, RT-PCR amplification was performed with forward primers for the respective V-region containing a 5’ restriction overhang (BssHII) and reverse primers encoding partial CDR3 (D-region) and 18-bases of FR4 sequence (J-region). (**c**) PCR amplification was performed using forward primers with restriction digestion sites (BssHII) and 3 nucleotides of the respective V-region, and reverse primers encoding complete FR4 sequences with a restriction site at the 3’ end (NheI/HindIII). Primers used in the first step RT-PCR and second step PCR amplifications are listed in Supplementary table 3. (**d**) The HC and LC genes were cloned into expression vectors for IgG and IgK chains. The corresponding paired HC and LC expression constructs were co-transfected into HEK293T cells for expression and production of recombinant monoclonal antibodies.


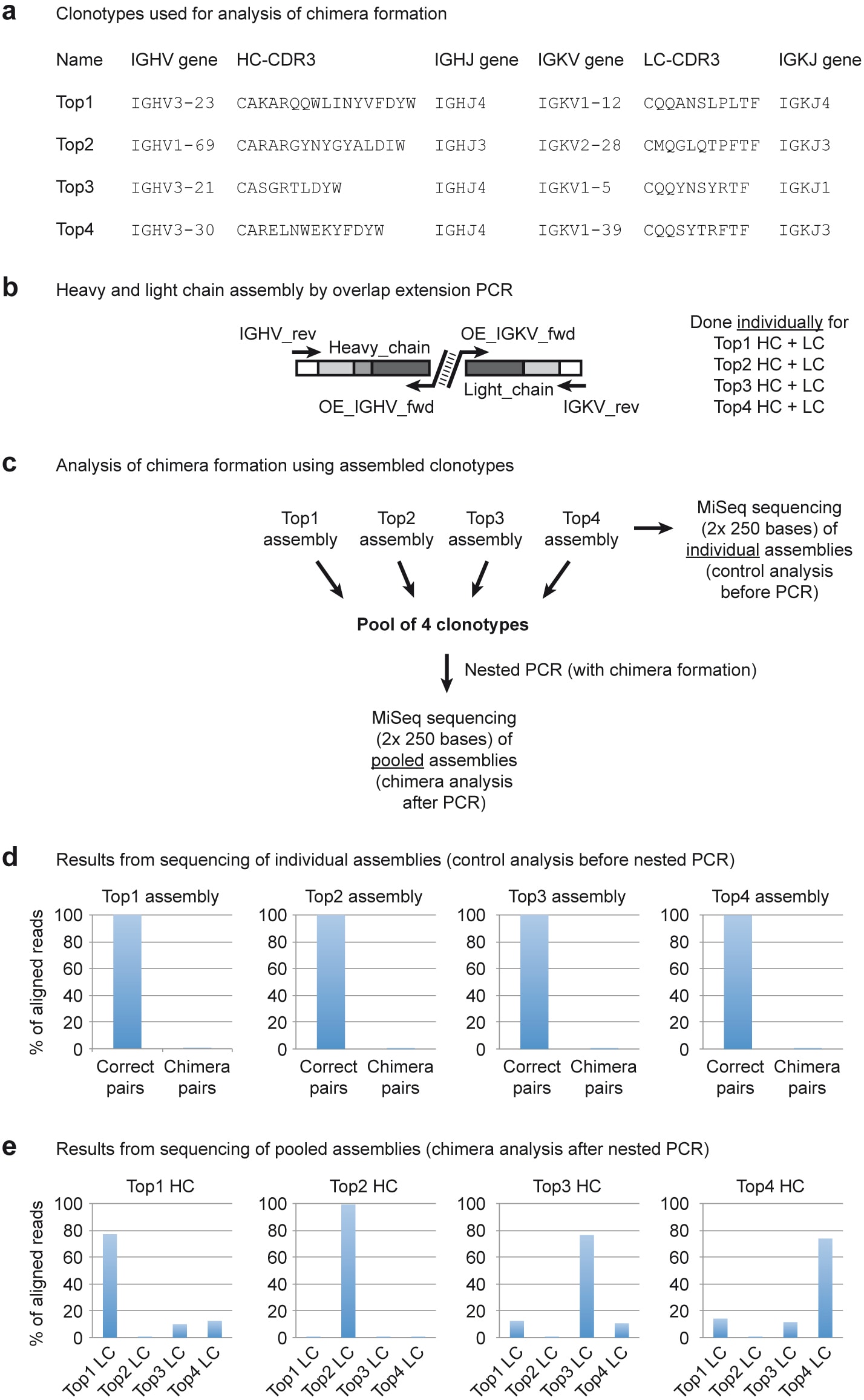


**Figure S6:** Analysis of chimera formation during the secondary nested PCR. (**a**) HC and LC plasmid DNA from Top1, Top2, Top3 and Top4 clonal pairs were used as templates for PCR amplification to generate four different defined HC-LC amplicons. (**b**) Overlap extension assembly PCR was performed individually for each clonal pair using IgG and IgK constant region primers. (**c**) The assembly products from all four clonotypes were pooled together to perform nested PCR amplification using IgG and IgK nested primers. Sequencing libraries were prepared from each individual assembly before nested PCR (as control for sequence purity) and from the pooled assemblies after nested PCR, followed by 2x250 base paired end sequencing on the Illumina MiSeq platform. (**d**) Analysis of the sequencing reads revealed >99.6% correct HC-LC pairs in the individual assemblies. Analysis of the reads from the pooled assemblies after nested PCR revealed 10-14% chimera formation among the Top1, Top3 and Top4 clonotypes, while the Top2 clonotype showed <0.3% chimeras with the other three clonotypes.

| **Immunization** | **Pre-immunization**  **(day 0)** | | **Post-Td booster immunization (day 7)** | |
| --- | --- | --- | --- | --- |
| Number of fresh CD19+ B cells used for preparation of microsomes | 1.5 million | | 1.5 million | |
| Isotype | IgM | IgG | IgM | IgG |
| rER microsomes corresponding to approximate number of B cells used for isotype-specific OE RT-PCR assembly | 462,000 | 462,000 | 462,000 | 462,000 |
| rER microsomes corresponding to approximate number of ARH-77 cells spiked to B cells for isotype-specific OE RT-PCR assembly | n.a. | 2,000 | n.a. | 2,000 |
| Raw sequencing read pairs (HC-LC) obtained from MiSeq 2x250 bases paired end sequencing | 5,860,554 | 5,185,375 | 5,356,219 | 4,979,748 |
| Total number of aligned HC-LC read pairs | 5,238,212 | 4,411,684 | 4,647,787 | 4,332,934 |
| Unique HC-LC pairs with ≥1 read per pair | 5,858 | 19,090 | 9,609 | 11,119 |
| HC-LC pairs with ≥2 reads per pair | 2,200 | 4,841 | 4,031 | 2,872 |

**Table S1:** Overview of IgM and IgG paired HC-LC repertoires pre-(day 0) and 7 days post-Td booster immunization.

| **Working concentration (nM)** | **Primer name** | **Primer sequence (5’-3’)** |
| --- | --- | --- |
| 375 | IgM_RT_rev | CACAGGAGACGAGGGGGAAA |
| 375 | IgG_RT_rev | AGGGYGCCAGGGGGAAGAC |
| 375 | IgK_RT_rev | GATGAAGACAGATGGTGCAG |
| 17.5 | Overlap_VH1/7_fwd | tcgtgcctatatccttactgactctgcGGCCTCAGTGAAGGTCTCCTGCAAG |
| 17.5 | Overlap_VH2_fwd | tcgtgcctatatccttactgactctgcGTCTGGTCCTACGCTGGTGAAACCC |
| 17.5 | Overlap_VH3_fwd | tcgtgcctatatccttactgactctgcCTGGGGGGTCCCTGAGACTCTCCTG |
| 17.5 | Overlap_VH4_fwd | tcgtgcctatatccttactgactctgcCTTCGGAGACCCTGTCCCTCACCTG |
| 17.5 | Overlap_VH5_fwd | tcgtgcctatatccttactgactctgcCGGGGAGTCTCTGAAGATCTCCTGT |
| 17.5 | Overlap_VH6_fwd | tcgtgcctatatccttactgactctgcTCGCAGACCCTCTCACTCACCTGTG |
| 7 | Overlap_VK1_fwd | gcagagtcagtaaggatataggcacgaGACATCCRGDTGACCCAGTCTCC |
| 7 | Overlap_VK2/4/6_fwd | gcagagtcagtaaggatataggcacgaGGGATATTGTGMTGACYCAGWCTCC |
| 7 | Overlap_VK3_fwd | gcagagtcagtaaggatataggcacgaGGAGAAATTGTRWTGACRCAGTCTCC |
| 7 | Overlap_VK5_fwd | gcagagtcagtaaggatataggcacgaGCAGAAACGACACTCACGCAGTCTC |
| 400 | IgM_nested | GGTTGGGGCGGATGCACTCC |
| 400 | IgG_nested | SGATGGGCCCTTGGTGGARGC |
| 400 | IgK_nested | CAGATGGTGCAGCCACAGTTC |

**Table S2:** Primer sequences used for overlap extension RT-PCR and nested PCR.

| **Primer name** | **Primer sequence (5’-3’)** |
| --- | --- |
| HC_VH1_FR1_Fwd | ATCTAAGCGCGCACTCCCAGGTCCAGCTKGTRCAGTCTGG |
| HC_VH3_FR1_Fwd | ATCTAAGCGCGCACTCCGAGGTGCAGCTGKTGGAGWCY |
| HC_VH4_FR1_Fwd | ATCTAAGCGCGCACTCCCAGGTGCAGCTGCAGGAGTCSG |
| LC_VK1_FR1_Fwd | ATATAAGCGCGCACTCCGACATCCRGDTGACCCAGTCTCC |
| LC_VK246_FR1_Fwd | ATATAAGCGCGCACTCCGATATTGTGMTGACBCAGWCTCC |
| LC_VK3_FR1_Fwd | ATATAAGCGCGCACTCCGAAATTGTRWTGACRCAGTCTCC |
| LC_VK5_FR1_Fwd | ATATAAGCGCGCACTCCGAAACGACACTCACGCAGTCTC |
| HC_Top1_CDR3_Rev | GACCAGGGTTCCCTGGCCCCAGTAGTCAAAGACATAGTTAATGAGCCACTGCTGTCTCGCTTTCGC |
| HC_Top2_CDR3_Rev | GACCATTGTCCCTTGGCCCCAGATATCAAGAGCATAACCATAATTGTATCCCCTAG |
| HC_Top3_CDR3_Rev | GACCAGGGTTCCCTGGCCCCAGTAATCCAACGTCCTCCCACTCGCACA |
| HC_Top4_CDR3_Rev | GACCAGGGTTCCCTGGCCCCAGTAGTCAAAGTACTTCTCCCAATTCAGCTCTCTCGC |
| HC_Top5_CDR3_Rev | CCAGATATCAAAAGCATTCCAGCTGGTACTATAGCCGACATTCCTATCTCTCGC |
| HC_Top6_CDR3_Rev | GACCATTGTCCCTTGGCCCCAGAGATCGAAAGGAACGAGAGCAGCTGGAACAACTAAAATATCGCGAGCCCCCCCTCT |
| HC_Top7_CDR3_Rev | GACCAGGTTTCCCTGGCCCCAGGGGTCGAACCAGTTCAGGGCGTCACGAATCGCTTTAGTTTCTCTCGC |
| HC_Top8_CDR3_Rev | GACCAGGGTTCCCTGGCCCCAGTAGTCAAAGTATCCGGCTCCGTGAACCATAATGGTATCTCTCGC |
| HC_Top9_CDR3_Rev | GACCAGGGTGCCACGGCCCCAGAGAGCGAAGTACCAGTAGGGGATGAGATAACCATTCAAAATATCGTAATTGAT |
| HC_Top10_CDR3_Rev | GACCATTGTCCCTTGGCCCCAGAGATCGAAAGGAACGAGAGCAGCTGGAACACCTAAAATATCGCGAGTCC |
| HC_Top11_CDR3_Rev | GACCAGACTTCCCTGGCCCCAGTGGTCAGAGTAGCCGACTCCCCTAACCATACGGGTATCTCTCGC |
| HC_Top12_CDR3_Rev | GACCAGGGTTCCCTGGCCCCAGAAGGAGTAGTAACCAGAAGTATTATAGTAATGAGTTGC |
| HC_Top13_CDR3_Rev | GACCACTGTCCCTTGGCCCCAGACATCAAAAGCATTCCAGCTGGTACTATAGCCGACATTCCTATCTCTCGC |
| HC_Top14_CDR3_Rev | GACCAGGGTTCCCTGGCCCCAGTAGTCAAAACTAGGTCGTCCCATAGCTGTATTCGGCCGACTTCTCGC |
| Known_HC_CDR3_Rev | GACCAGGGTTCCCTGGCTCCAGGGGTCGAACCAGTTGTCAGCCTGTCTC |
| LC_Top1_CDR3_Rev | CACCTTGGTCCCTCCGCCGAAAGTGAGCGGGAGACTGTTAGCCTGTTG |
| LC_Top2_CDR3_Rev | CACTTTGGTCCCAGGGCCGAAAGTGAAAGGAGTTTGCAGACCTTGCAT |
| LC_Top3_CDR3_Rev | CACCTTGGTCCCTTGGCCGAACGTCCGATAACTATTATACTGTTGGCA |
| LC_Top4_CDR3_Rev | CACCTTGGTCCCAGGGCCGAAAGTGAATCGGGTGTAACTCTGTTG |
| LC_Top5_CDR3_Rev | CACCTTGGTCCCTTGGCCGAACGTTCGAGAATAACTATTATACTGTTG |
| LC_Top6_CDR3_Rev | CAGTCGTGTCCCTTGGCCGAAGGTGATCGGAATACCATAACATTGCTG |
| LC_Top7_CDR3_Rev | CACCTTGGTCCCCCCGCCGAAAGTGAAGACGAAACTGTAACTCTGTAG |
| LC_Top8_CDR3_Rev | CACCTTGGTCCCTCCGCCGAAAGTGAGCGGGGTACTGTAACTCTGTTG |
| LC_Top9_CDR3_Rev | CACTTTGGTCCCAGGGCCGAAAGTGAACCTCGACTGTTG |
| LC_Top10_CDR3_Rev | CAGTCGTGTCCCTTGGCCGAAGGTGATCGGAATACCATAACATTGCTG |
| LC_Top11_CDR3_Rev | CACCTTGGTCCCTCCGCCGAAAGTGAGCGGGAAACTGTTAGCCTGCTG |
| LC_Top12_CDR3_Rev | CAGCTTGGTCCCCTGGCCAAAAGTGTACAGGGGACTGGAAGTCTGTTG |
| LC_Top13_CDR3_Rev | CACCTTGGTCCCTTGGCCGAACGTTCGAGAATAACTATTATACTGTTG |
| LC_Top14_CDR3_Rev | CACCTTGGTCCCTCCGCCGAAAGTGAGCGGGGTACTGTAACTCTGTTG |
| Known_LC_CDR3_Rev | CAGCTTGGTCCCCTGGCCAAAAGTTAGAGGGAAATCATCATGTTGTAG |
| HC_VH1_RD_FR1_Fwd | ATCTAAGCGCGCACTCCCAG |
| HC_VH3_RD_FR1_Fwd | ATCTAAGCGCGCACTCCGAG |
| LC_VK1_RD_FR1_Fwd | ATATAAGCGCGCACTCCGAH |
| LC_VK3_RD_FR1_Fwd | ATATAAGCGCGCACTCCGAA |
| HC_Top2_RD_FR4_Rev | AAATTTGCTAGCGCTCGAGTGAAGAGACGGTGACCATTGTCCCTTGGCCCC |
| HC_Top1_Top3_Top4_  Top8_Top12_RD_FR4_Rev | AAATTTGCTAGCGCTCKAGGAGGAGACGGWGACCAGGGTTCCCTGGCC |
| HC_Top5_RD_FR4_Rev | AAATTTGCTAGCGCTCGAGGAAGAGACGGTGACCACTGTCCCTTGGCCCCAGATATCAAAAGCATT |
| HC_Top6_Top10_FR4_  RD_Rev | AAATTTGCTAGCGCTCGAGGAAGAGATGGTGACCATTGTCCCTTGGCC |
| HC_Top7_RD_FR4_Rev | AAATTTGCTAGCGCTCGAGGAGGAGACGGTGACCAGGTTTCCCTGGCC |
| HC_Top9_RD_FR4_Rev | AAATTTGCTAGCGCTCGAGGAGGAGACAGCGACCAGGGTGCCACGGCC |
| HC_Top11_RD_FR4_Rev | AAATTTGCTAGCGCTCGAGGAGGAGACGGTGACCAGACTTCCCTGGCC |
| HC_Top13_RD_FR4_Rev | AAATTTGCTAGCGCTCGAGGAAGAGACGGTGACCACTGTCCCTTGGCC |
| HC_Top14_RD_FR4_Rev | AAATTTGCTAGCGCTCGAGGAGGAGACGGTGACCAGGGTTCCCTGGCC |
| HC_Known_RD_FR4_Rev | AAATTTGCTAGCGCTCGAGGAGGAGACGGTGACCAGGGTTCCCTGGCT |
| LC_Top2_RD_FR4_Rev | ATATTAAAGCTTAAGCTTTTTGATATCCACTTTGGTCCCAGGGCC |
| LC_Top3_Top5_Top13_  RD_FR4_Rev | ATTATAAAGCTTTTTGATTTCCACCTTGGTCCCTTGGCC |
| LC_Top1_Top8_Top11_  Top14_RD_FR4_Rev | ATTATAAAGCTTTTTGATCTCCACCTTGGTCCCTCCGCC |
| LC_Top4_RD_FR4_Rev | ATTATAAAGCTTTTTGATATCCACCTTGGTCCCAGGGCC |
| LC_Top6_RD_FR4_Rev | ATTATAAAGCTTTTTAATCTCCAGTCGTGTCCCTTGGCC |
| LC_Top7_RD_FR4_Rev | ATTATAAAGCTTTCTGATCTCCACCTTGGTCCCCCCGCC |
| LC_Top9_RD_FR4_Rev | ATTATAAAGCTTTTTGATATCCACTTTGGTCCCAGGGCC |
| LC_Top10_RD_FR4_Rev | ATTATAAAGCTTTTTAATCTCCAGTCGTGTCCCTTGGCC |
| LC_Top12_RD_FR4_Rev | ATTATAAAGCTTTTTGATCTCCASCTTGGTCCCCTGGCC |
| LC_known_RD_FR4_Rev | ATTATAAAGCTTTTTGATGTCCAGCTTGGTCCCCTGGCC |

**Table S3:** HC and LC RT-PCR amplification primers used for fifteen selected clonotypes. HC and LC V-region forward primers with restriction digestion sites are denoted by “FR1_Fwd” and CDR3 clonotype-specific reverse primers are denoted by “CDR3_Rev”. Primers used in second step PCR amplification are denoted by “RD_FR1_Fw” for forward primers and “RD_FR4_Rev” for reverse primers.
